# Use and Content of Primary Care Office-Based vs Telemedicine Care Visits During the COVID-19 Pandemic in the US

**DOI:** 10.1001/jamanetworkopen.2020.21476

**Published:** 2020-10-02

**Authors:** G. Caleb Alexander, Matthew Tajanlangit, James Heyward, Omar Mansour, Dima M. Qato, Randall S. Stafford

**Affiliations:** 1 Center for Drug Safety and Effectiveness, Johns Hopkins Bloomberg School of Public Health, Baltimore, Maryland; 2 Department of Epidemiology, Johns Hopkins Bloomberg School of Public Health, Baltimore, Maryland; 3 Division of General Internal Medicine, Johns Hopkins Medicine, Baltimore, Maryland; 4 Student, Johns Hopkins University, Baltimore, Maryland; 5 Monument Analytics, Baltimore, Maryland; 6 Department of Pharmacy Systems, Outcomes, and Policy, University of Illinois at Chicago, Chicago; 7 Stanford Prevention Research Center, Stanford University, Palo Alto, California

## Abstract

**Importance:**

Little is known about the association between the coronavirus disease 2019 (COVID-19) pandemic and the level and content of primary care delivery in the US.

**Objective:**

To quantify national changes in the volume, type, and content of primary care delivered during the COVID-19 pandemic, especially with regard to office-based vs telemedicine encounters.

**Design, Setting, and Participants:**

Analysis of serial cross-sectional data from the IQVIA National Disease and Therapeutic Index, a 2-stage, stratified nationally representative audit of outpatient care in the US from the first calendar quarter (Q1) of 2018 to the second calendar quarter (Q2) of 2020.

**Main Outcomes and Measures:**

Visit type (office-based or telemedicine), overall and stratified by patient population and geographic region; assessment of blood pressure or cholesterol measurement; and initiation or continuation of prescription medications.

**Results:**

In the 8 calendar quarters between January 1, 2018, and December 31, 2019, between 122.4 million (95% CI, 117.3-127.5 million) and 130.3 million (95% CI, 124.7-135.9 million) quarterly primary care visits occurred in the US (mean, 125.8 million; 95% CI, 121.7-129.9 million), most of which were office-based (92.9%). In 2020, the total number of encounters decreased to 117.9 million (95% CI, 112.6-123.2 million) in Q1 and 99.3 million (95% CI, 94.9-103.8 million) in Q2, a decrease of 21.4% (27.0 million visits) from the average of Q2 levels during 2018 and 2019. Office-based visits decreased 50.2% (59.1 million visits) in Q2 of 2020 compared with Q2 2018-2019, while telemedicine visits increased from 1.1% of total Q2 2018-2019 visits (1.4 million quarterly visits) to 4.1% in Q1 of 2020 (4.8 million visits) and 35.3% in Q2 of 2020 (35.0 million visits). Decreases occurred in blood pressure level assessment (50.1% decrease, 44.4 million visits) and cholesterol level assessment (36.9% decrease, 10.2 million visits) in Q2 of 2020 compared with Q2 2018-2019 levels, and assessment was less common during telemedicine than during office-based visits (9.6% vs 69.7% for blood pressure; *P* < .001; 13.5% vs 21.6% for cholesterol; *P* < .001). New medication visits in Q2 of 2020 decreased by 26.0% (14.1 million visits) from Q2 2018-2019 levels. Telemedicine adoption occurred at similar rates among White individuals and Black individuals (19.3% vs 20.5% of patient visits, respectively, in Q1/Q2 of 2020), varied by region (low of 15.1% of visits [East North Central region], high of 26.8% of visits [Pacific region]), and was not correlated with regional COVID-19 burden.

**Conclusions and Relevance:**

The COVID-19 pandemic has been associated with changes in the structure of primary care delivery, with the content of telemedicine visits differing from that of office-based encounters.

## Key Points

**Question:**

Is there a quantifiable association between the coronavirus disease 2019 (COVID-19) pandemic and the volume, type, and content of primary care encounters in the US?

**Findings:**

In this cross-sectional analysis of the US National Disease and Therapeutic Index audit of more than 125.8 million primary care visits in the 10 calendar quarters between quarter 1 of 2018 and quarter 2 of 2020, primary care visits decreased by 21.4% during the second quarter of 2020 compared with the average quarterly visit volume of the second quarters of 2018 and 2019. Evaluations of blood pressure and cholesterol levels decreased owing to fewer total visits and less frequent assessment during telemedicine encounters.

**Meaning:**

The COVID-19 pandemic was associated with changes in the structure of primary care delivery during the second quarter of 2020, with the content of telemedicine visits differing from that of office-based encounters.

## Teaser

This cross-sectional study uses data from the National Disease and Therapeutic Index to quantify changes in volume and type of primary care visits and changes in visit content associated with the COVID-19 pandemic in the US.

## Introduction

Since February 2020, the coronavirus disease 2019 (COVID-19) pandemic has been associated with more than 4.4 million cases and 150 000 deaths in the US, as well as widespread social and economic changes.^1^ While the ultimate health care system impacts of the pandemic remain uncertain, many early health care consequences associated with the pandemic have been noted, ranging from postponement of elective care to permanent clinic and hospital closures.^2,3^

Early reports suggested that substantial increases could be expected in the delivery of telemedicine, or remote clinical services, during the first few months of the pandemic in the US,^4,5^ owing to concern regarding the potential for workplace transmission of COVID-19, the implementation of social distancing policies, and the redeployment of health care personnel. A more recent update indicated that the delivery of telemedicine increased during mid-April and has since subsided modestly, although levels remain substantially higher than before the pandemic.^6^ These changes, which have been accompanied by changes in federal^7,8^ and state^9,10^ guidance and reimbursement, have occurred in the context of structural and social factors^11,12^ hindering widespread telemedicine adoption.

Investigations of telemedicine during the pandemic, while yielding insights, have generally been based on small or nonrepresentative samples and limited to analyses of the frequency of such encounters rather than descriptions of their content.^4,5,6,13^ We quantified national changes in the volume and type of primary care associated with the COVID-19 pandemic. In addition to characterizing blood pressure and cholesterol measurement and initiation or continuation of prescription medicines for hypertension and dyslipidemia, we explored variance in telemedicine use across different patient populations and geographic regions of the US.

## Methods

### Data

We used the IQVIA National Disease and Therapeutic Index to conduct a cross-sectional analysis, focusing on the period from the first quarter (Q1) of 2018 through the second quarter (Q2) of 2020. The National Disease and Therapeutic Index is a nationally representative audit of outpatient practice in the US.^14,15^ Other studies^16,17,18^ have compared the National Disease and Therapeutic Index with the National Ambulatory Medical Care Survey, a nationally representative audit conducted by the National Center for Health Statistics, and found that the surveys yielded substantively comparable estimates of outpatient care. Using a 2-stage, stratified sampling design, the National Disease and Therapeutic Index audit is based on a sample derived from the American Medical Association and the American Osteopathic Association.^19^ Data are collected from approximately 4000 physicians during each calendar quarter, during which participants complete a form for 2 consecutive days documenting each patient encounter, including demographic information, diagnoses, and treatment provided. Diagnostic information is reported using a system similar to the *International Classification of Diseases, Ninth Revision*. The original development of the National Disease and Therapeutic Index was limited to the contiguous US because of statistical sampling and design considerations; as a result, physician reporting from Alaska and Hawaii are excluded. Each encounter is also classified based on site of care, including office-based, hospital-based, telemedicine, or other (such as home, nursing facility, or other institutional setting). Clinician-reported demographic information about individuals, such as patient age, sex, and race, were also assessed. Data were then weighted to provide nationally representative estimates as well as estimates that are representative across 8 geographic regions (Pacific, East South Central, West South Central, West North Central, Mountain, South Atlantic, East North Central, and New England and Mid-Atlantic) (eTable 1 in the Supplement). This study was deemed exempt from institutional review board review by the Johns Hopkins Bloomberg School of Public Health per regulations found at 45 CFR 46. This study followed the Strengthening the Reporting of Observational Studies in Epidemiology (STROBE) reporting guideline for cross-sectional studies.^20^

We restricted our analysis to primary care visits, defined as those accounted for by the fields of internal medicine, pediatrics, geriatrics, general practice, and family practice. For cases in which we specifically assessed pharmaceutical prescribing (eg, initiations of pharmacologic therapy for specific diseases), we examined treatment visit, defined as a patient encounter for a specific diagnosis in which a pharmacologic treatment was initiated or continued. Except where depicted otherwise, we excluded the approximately 3% to 4% of hospital-based visits and 1% to 2% of visits taking place in other settings (home, nursing home, and unspecified sites of care) each calendar quarter.

Our analysis of telemedicine use across geographic regions included an examination of how such use varied by COVID-19 burden, expressed as the rate of COVID-19 fatalities per 100 000 individuals. To estimate this rate, we summed COVID-19 deaths within each national region as of July 28, 2020,^21^ and divided this number by the total population within that region.^22^

### Statistical Analysis

We used descriptive statistics to perform our analysis. Our main outcomes were visit type, assessment of blood pressure or cholesterol measurement, and initiation or continuation of prescription medicines. First, we extracted the total number of visits between January 1, 2018, and June 30, 2020, and plotted these numbers over time to examine general trends and to assess for inflection points and outliers. Next, we aggregated these visits by calendar quarter. We then characterized the distribution of visits, stratified by encounter type, across visit characteristics of interest, such as patient age, sex, race, and type of insurance. We limited our analysis of telemedicine use by race to Black race and White race given racial disparities in health care. We calculated a Pearson correlation coefficient to examine the association between telemedicine use and COVID-19 burden across 8 geographic regions. We used standardized errors to estimate 95% CIs. Analyses used 2-tailed, unpaired testing. A threshold of *P* < .05 was used to establish statistical significance. Statistical analyses were conducted using Stata software, version 15 (StataCorp LLC).

## Results

### Trends in Primary Care Visits by Encounter Type

In the 8 calendar quarters between January 1, 2018, and December 31, 2019, between 122.4 million (95% CI, 117.3-127.5 million) and 130.3 million (95% CI, 124.7-135.9 million) quarterly primary care visits occurred in the US (mean, 125.8 million; 95% CI, 121.7-129.9 million) (Table 1) (eFigure in the Supplement). In 2020, the total number of encounters decreased to 117.9 million (95% CI, 112.6-123.2 million) in Q1 and 99.3 million (95% CI, 94.9-103.8 million) in Q2, a decrease of 21.4% (27.0 million visits) from the average number of Q2 encounters in 2018 and 2019.

**Table 1.** Trends in Primary Care by Visit Type, 2018-2020^a^

Variable	No., in thousands (%)									
	2018				2019				2020	
	Q1	Q2	Q3	Q4	Q1	Q2	Q3	Q4	Q1	Q2
Total visits, No. (95% CI)	124 919 (119 697-130 141)	122 402 (117 286-127 518)	124 115 (118 927-129 303)	125 854 (120 593-131 115)	127 298 (121 786-132 810)	130 297 (124 655-135 939)	128 225 (122 673-133 777)	123 180 (117 846-128 514)	117 944 (112 648-123 240)	99 326 (94 866-103 786)
Office-based	116 614 (93.35)	114 249 (93.34)	115 780 (93.28)	117 219 (93.14)	117 741 (92.49)	121 344 (93.13)	117 936 (91.98)	114 176 (92.69)	105 911 (89.80)	58 668 (59.07)
Hospital-based	4126 (3.30)	3786 (3.09)	4189 (3.38)	4376 (3.48)	4949 (3.89)	4736 (3.63)	4951 (3.86)	4366 (3.54)	4229 (3.59)	3353 (3.38)
Telemedicine	1398 (1.12)	1194 (0.98)	1109 (0.89)	1363 (1.08)	1355 (1.06)	1611 (1.24)	1406 (1.10)	1369 (1.11)	4794 (4.07)	35 044 (35.28)
Other^b^	2780 (2.23)	3172 (2.59)	3037 (2.45)	2897 (2.30)	3253 (2.56)	2606 (2.00)	3933 (3.07)	3267 (2.65)	3009 (2.55)	2262 (2.28)

Abbreviation: Q, quarter.

^a^ Source: IQVIA National Disease and Therapeutic Index, 2018-2020.^19^

^b^ Other includes home, nursing home, and unspecified sites of care.

Most primary care encounters in 2018-2019 were office-based (92.9%). Office-based visits decreased from a mean of 116.9 million (95% CI, 111.6-122.1 million) for the average quarterly visit volume in 2018-2019 to 105.9 million (95% CI, 101.2-110.7 million) in Q1 of 2020, then 58.7 million (95% CI, 55.3-62.1 million) in Q2 of 2020, a decrease of 50.2% (59.1 million visits) compared with Q2 2018-2019 levels. By contrast, telemedicine visits increased from 1.1% of visits in 2018-2019 to 4.1% in Q1 of 2020 and 35.3% of visits in Q2 of 2020.

### Primary Care Visits by Race and Other Patient Characteristics

Table 2 presents the use of office-based and telemedicine visits stratified by patient race. For example, during the first 2 quarters of 2018, there were 158.8 million (95% CI, 152.1-165.4 million) patient visits among White individuals (85.6% of visits among White or Black individuals) and 26.7 million (95% CI, 24.6-28.7 million) patient visits among Black individuals (14.4%). Visits for Black individuals accounted for between 14.4% and 17.4% of visits of the period examined, and increases in telemedicine visits were similar among White individuals and Black individuals, with telemedicine visits accounting for 19.3% of 2020 treatment visits among White individuals and 20.5% of those of Black individuals.

**Table 2.** Primary Care Office-Based and Telemedicine Visits by Patient Race, 2018-2020^a^

Variable	No., in thousands (%)^b^								
	Both White and Black individuals			White individuals			Black individuals		
	2018 (Q1/Q2)	2019 (Q1/Q2)	2020 (Q1/Q2)	2018 (Q1/Q2)	2019 (Q1/Q2)	2020 (Q1/Q2)	2018 (Q1/Q2)	2019 (Q1/Q2)	2020 (Q1/Q2)
All visits, No. (95% CI)	185 457 (177 705-193 209)	189 595 (181 386-197 804)	161 063 (153 831-168 295)	158 784 (152 147-165 421)	160 210 (153 273-167 147)	133 042 (127 068-139 016)	26 672 (24 648-28 696)	29 385 (27 075-31 695)	28 021 (25 737-30 305)
All visits, %	NA	NA	NA	85.6	84.5	82.6	14.4	15.5	17.4
Office-based	183 255 (98.8)	187 065 (98.7)	129 658 (80.5)	156 795 (98.7)	157 874 (98.5)	107 389 (80.7)	26 460 (99.2)	29 191 (99.3)	22 269 (79.5)
Telemedicine	2201 (1.2)	2530 (1.3)	31 405 (19.5)	1989 (1.3)	2336 (1.5)	25 653 (19.3)	212 (0.8)	194 (0.7)	5752 (20.5)

Abbreviations: NA, not applicable; Q, quarter.

^a^ Source: IQVIA National Disease and Therapeutic Index, 2018-2020.^19^

^b^ Values represent cumulative visits for each period.

eTable 2 in the Supplement presents the distribution of office-based and telemedicine visits stratified by patient age, sex, and insurance type (commercial, Medicaid, or other). Whereas individuals aged 19 to 35 years and aged 36 to 55 years accounted for 12.4% and 19.8% of office-based visits, respectively, in Q1/Q2 of 2020, they accounted for 17.8% and 26.1% of telemedicine visits, respectively, during this period, indicating substantial adoption of telemedicine compared with their younger or older counterparts (15.6% of telemedicine visits were individuals aged <19 years and 15.2% and 25.3% of visits were individuals aged 56-65 years and ≥66 years, respectively). Commercially insured visits accounted for an average of 60.3% of office-based visits for Q1 and Q2 of 2020 and an average of 57.3% of telemedicine visits for Q1 and Q2 of 2020.

### Geographic Variation in Telemedicine Use

Table 3 and the Figure depict 2020 primary care office-based and telemedicine visits in the US stratified by geographic region. For example, during the first 2 quarters of 2020, there were 39.6 million (95% CI, 36.8-42.4 million) visits in the Pacific region (Washington, Oregon, and California). Of these visits, 10.6 million (95% CI, 9.2-12.1 million) (26.8%) were telemedicine encounters. The proportion of visits delivered by telemedicine varied from a low of 15.1% in the East North Central region (Wisconsin, Michigan, Illinois, Indiana, and Ohio) to a high of 26.8% in the Pacific region. Table 3 also presents the burden of the pandemic across these regions, with a case fatality rate that ranged from 19.90 to 124.91 per 100 000 individuals. There was no association between the use of telemedicine and the pandemic burden across geographic regions (*r* = 0.004; *P* = .99).

**Table 3.** Primary Care Office-Based and Telemedicine Visits by Geographic Region and COVID-19 Burden, First 2 Quarters of 2020^a^

Geographic region	COVID-19 death rate (per 100 000 individuals)	Visits, No. in thousands (95% CI)		Telemedicine % of total
		Office-based	Telemedicine	
Pacific	19.90	29 000 (26 637-31 364)	10 631 (9158-12 104)	26.8
East South Central	24.23	9033 (7718-10 348)	1928 (1386-2470)	17.6
West South Central	25.30	14 792 (12 742-16 842)	2908 (2215-3601)	16.4
West North Central	21.11	8807 (7525-10 089)	1711 (1230-2192)	16.3
Mountain	27.62	9959 (8579-11 339)	2394 (1722-3066)	19.4
South Atlantic	29.65	36 238 (33 676-38 800)	8146 (6892-9400)	18.4
East North Central	44.97	27 402 (25 169-29 635)	4856 (3929-5783)	15.1
New England and Mid-Atlantic	124.91	29 349 (26 957-31 741)	7263 (6074-8452)	19.8
All regions	45.24	164 579 (157 189-171 969)	39 838 (37 021-42 655)	19.5

Abbreviation: COVID-19, coronavirus disease 2019.

^a^ Source: IQVIA National Disease and Therapeutic Index, 2018-2020.^19^

**Figure.**
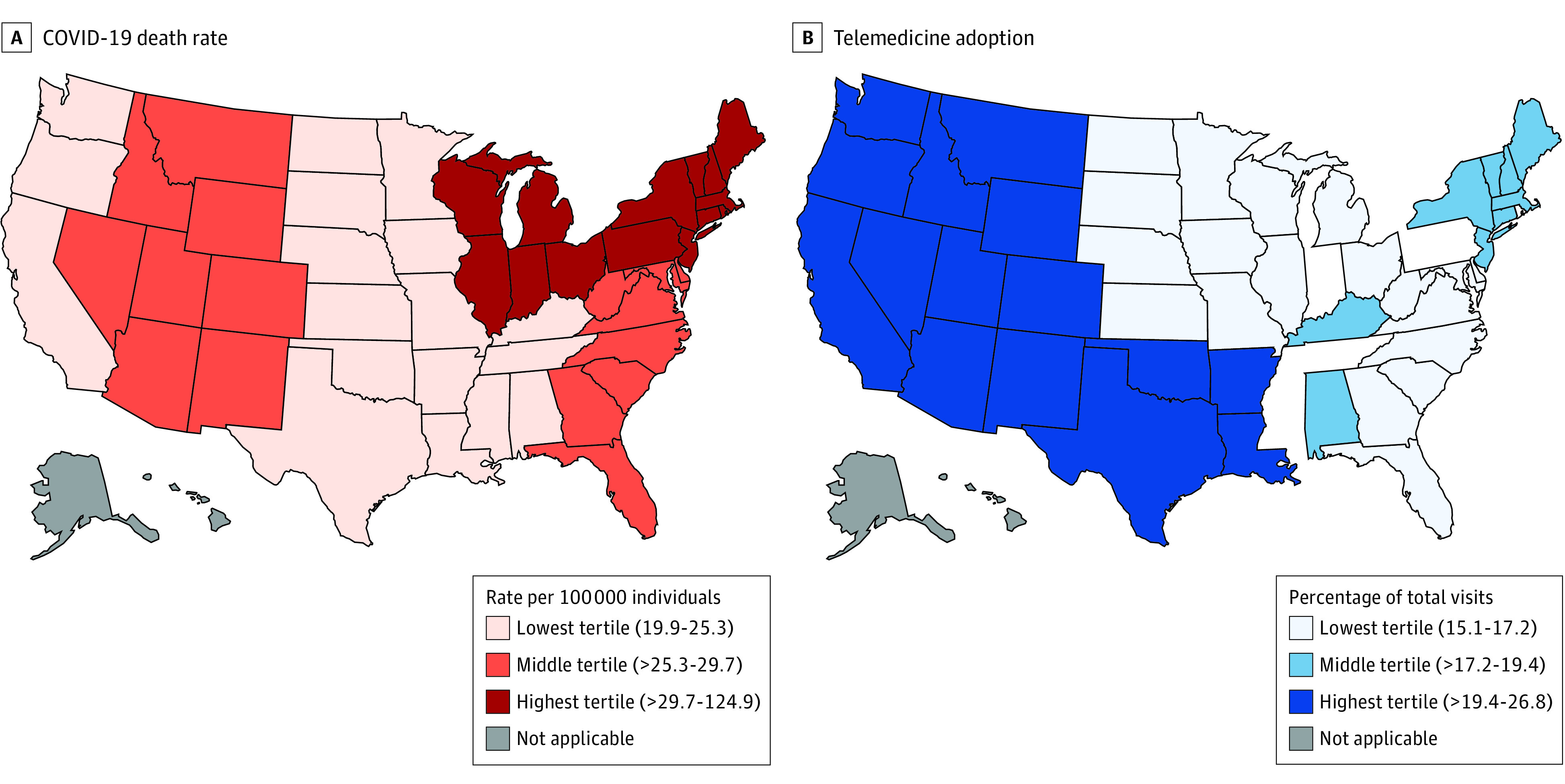
Geographic Variation in COVID-19 Burden and Telemedicine Adoption in the First 2 Quarters of 2020 COVID-19 indicates coronavirus disease 2019.

### Features of Primary Care Office-Based and Telemedicine Visits

Table 4 and eTable 3 in the Supplement characterize the content of primary care visits over time, showing reductions in care assessment. For example, blood pressure was assessed in an estimated 88.7 million (95% CI, 84.6-92.7 million) Q2 visits in 2018/2019, 75.9 million (95% CI, 72.0-79.7 million) visits in Q1 of 2020, and 44.2 million (95% CI, 41.1-47.4 million) visits in Q2 of 2020, reflecting a 50.1% decrease (44.4 million visits) from Q2 2018-2019 levels. The absolute number of cholesterol assessments decreased 36.9% over the same period (10.2 million visits), while reductions in the absolute number of office-based and telemedicine visits with the initiation (26.0%) or continuation (8.9%) of new medicines were also noted.

**Table 4.** Content of Primary Care Office-Based and Telemedicine Visits, 2018-2020^a^

Variable	No., in thousands (%)			% Change (2020 Q2 vs 2018-2019 Q2)^b^
	2018-2019 (Q2)	2020 (Q1)	2020 (Q2)	
Total visits, No. (95% CI)	119 199 (114 038-124 360)	110 705 (105 734-115 676)	93 712 (89 270-98 154)	−21.4
Blood pressure recorded	88 675 (74.4)	75 852 (68.5)	44 229 (47.2)	−50.1
Cholesterol assessed	27 617 (23.2)	22 803 (20.6)	17 413 (18.5)	−36.9
New medicines initiated	54 142 (45.4)	51 773 (46.8)	40 079 (42.8)	−26.0
Medicines continued	38 024 (31.9)	35 541 (32.1)	34 621 (36.9)	−8.9
New treatment visits				
Hypertension	3414 (2.9)	2714 (2.5)	2078 (2.2)	−39.1
Diabetes	1408 (1.2)	1226 (1.1)	1177 (1.3)	−16.4
High cholesterol	1274 (1.1)	1326 (1.2)	926 (1.0)	−27.3
Asthma	1266 (1.1)	1146 (1.0)	635 (0.7)	−49.8
Depression	193 (0.2)	157 (0.1)	149 (0.2)	−22.8
Insomnia	396 (0.3)	437 (0.4)	299 (0.3)	−24.5

Abbreviation: Q, quarter.

^a^ Values represent average second quarter visit volume (2018-2019) and quarterly visit volume (2020 Q1 and 2020 Q2).

^b^ Percentage change depicts a comparison of the second quarter of 2020 (2020 Q2) with average volume of 2018 Q2 and 2019 Q2 combined.

One reason for this finding was that assessment of blood pressure and cholesterol was statistically significantly less common among telemedicine than among office-based visits. For example, of 58.7 million (95% CI, 55.3-62.1 million) Q2 2020 office-based visits, 69.7% had a blood pressure recorded compared with 9.6% of telemedicine visits during the same time period (*P* < .001). Cholesterol was also less commonly assessed during telemedicine than during office-based visits (13.5% vs 21.6% of Q2 2020 visits; *P* < .001). New prescription medications were ordered similar proportions of Q2 2020 telemedicine and office-based visits (39.3% vs 44.9%), but absolute numbers across these visit types decreased from 54.1 million (95% CI, 50.8-57.5 million) in Q2 2018/2019 to 51.8 million (95% CI, 48.5-55.1 million) in Q1 of 2020 and 40.1 million (95% CI, 37.2-42.9 million) in Q2 of 2020, representing a 26.0% decrease from Q2 2018-2019 volume.

## Discussion

While the COVID-19 pandemic has impacted health care delivery in many ways, little is known regarding how the volume, site, and content of primary care in the US has changed. We used a nationally representative audit of outpatient care to characterize primary care delivery in the US between 2018 and Q2 of 2020. The pandemic has been associated with a more than 25% decrease in primary care volume, which has been offset in part by increases in the delivery of telemedicine, which accounted for 35.28% of encounters during the second quarter of 2020. Despite the increased use of telemedicine, its uptake has varied across the continental US and has not been correlated, at a regional level, with COVID-19 burden. Overall, the pandemic has been associated with marked reductions in the primary care assessment of cardiovascular risk factors such as blood pressure and cholesterol levels, owing to decreased total visit volume and less frequent assessment during telemedicine visits than during office-based visits. These findings are notable because little is known about the association between primary care delivery and the COVID-19 pandemic and because the pandemic has generated interest in telemedicine as a means to safely deliver primary care.

Our analysis was based on an assessment at a single point, and the degree to which the COVID-19 pandemic may be associated with permanent increases in the use of telemedicine remains to be seen. Historically, limited reimbursement, interstate licensure requirements, and patient and clinician factors have slowed the uptake and adoption of telemedicine.^12,23^ In response to the pandemic, US federal and state agencies and other stakeholders have modified policies and procedures, such as the Centers for Medicare & Medicaid Services provision of telehealth waivers for providers,^24^ to allow greater use of telemedicine to support remote clinical encounters.^25,26^ We did not find a correlation between regional COVID-19 burden and telemedicine adoption, suggesting that other factors may account for regional differences in the uptake of this mode of health care. In addition, whether the federal and state rules and regulations that have been modified will be made permanent and whether the current embrace of telemedicine by patients and clinicians will endure remain unknown.^27^

If substantial primary care volume continues to be delivered using telemedicine, a focus on the content and quality of such encounters is inevitable.^28^ Despite findings in a systematic review of 86 articles demonstrating the feasibility and acceptance of telemedicine for use in primary care, to our knowledge, relatively few rigorous comparisons of clinical outcomes in office-based vs telemedicine encounters have been performed.^29^ Our finding that such visits were less likely to include blood pressure or cholesterol assessments underscores the limitation of telemedicine, at least in its current form, for an important component of primary care prevention and chronic disease management.

Middle-aged individuals and those who were commercially insured were more likely to adopt telemedicine during the pandemic than their counterparts with other or no insurance. This difference may be due in part to the perceived elective or deferrable nature of visits among children^30^ and greater familiarity with telemedicine technology among middle-aged than among older adults.^31^ We did not find substantial differences in telemedicine use by payer type, and, contrary to our expectations and evidence of a digital divide,^32^ we did not find evidence of a racial disparity in telemedicine use when examining the frequency of telemedicine encounters as a proportion of a patient visits among Black versus White individuals.

### Limitations

This study has limitations. First, both the COVID-19 pandemic and health system response continue to evolve, and our analyses reflect the provision of care at a single point in time. Second, as with any outpatient audit, the data that we used were subject to measurement error, although prior analyses have yielded estimates comparable to those from the National Ambulatory Medical Care Survey. Third, factors such as patient race are complex multidimensional constructs, and our assessment of race in this context provides a limited window through which to understand how telemedicine adoption may vary across different populations.^33,34^ Fourth, our data did not allow us to examine more granular geographic associations between COVID-19 burden and telemedicine adoption. Last, we considered the assessment of 2 important cardiovascular risk factors, but many other dimensions of primary care might also be compared between office-based and telemedicine encounters, and we did not attempt to assess the association between primary care encounter type and overall quality of care.

## Conclusions

More than 4 months after the US Department of Health and Human Services declared a public health emergency, widespread economic and social changes in the US during the COVID-19 pandemic are still occurring. The pandemic has been associated with substantial decreases in primary care delivery, despite large increases in the use of telemedicine, which accounted for fewer than 2% of primary care visits during 2019 yet more than 35% of visits during Q2 of 2020. Evaluations of cardiovascular risk factors such as blood pressure and cholesterol have decreased, owing to fewer total visits and less frequent assessment during telemedicine encounters. Thus, the COVID-19 pandemic has been associated with changes in the structure of primary care, with the content of telemedicine visits differing from that of office-based encounters.

## Notes

Supplement. **eTable 1.** States Comprising IQVIA Subnational Regions in the National Disease and Therapeutic Index **eTable 2.** Characteristics of Primary Care Office-based and Telemedicine Visits, 2018-2020 (in thousands) **eTable 3.** Content of Primary Care Office-based and Telemedicine Visits, 2018-2020 (in thousands) **eFigure.** Trends in Primary Care Stratified by Visit Type, 2018-2020
