# Supplementary material for: Use and Content of Primary Care Office-Based vs Telemedicine Care Visits During the COVID-19 Pandemic in the US
Source: JAMA Netw Open. 2020 Oct 2;3(10):e2021476. doi: 10.1001/jamanetworkopen.2020.21476 (PMC7532385; doi:10.1001/jamanetworkopen.2020.21476)
Supplement: Supplement. — eTable 1. States Comprising IQVIA Subnational Regions in the National Disease and Therapeutic Index eTable 2. Characteristics of Primary Care Office-based and Telemedicine Visits, 2018-2020 (in thousands) eTable 3. Content of Primary Care Office-based and Telemedicine Visits, 2018-2020 (in thousands) eFigure. Trends in Primary Care Stratified by Visit Type, 2018-2020 [file jamanetwopen-e2021476-s001.pdf]

Supplementary Online Content

Alexander GC, Tajanlangit M, Heyward J, Mansour O, Qato DM, Stafford RS. Use and content of primary care office-based vs telemedicine care visits during the COVID-19 pandemic in the US. *JAMA Netw Open*. 2020;3(9):e2021476. doi:10.1001/jamanetworkopen.2020.21476

eTable 1. States Comprising Subnational Regions in the IQVIA National Disease and Therapeutic Index
eTable 2. Characteristics of Primary Care Office-based and Telemedicine Visits, 2018-2020 (in thousands)
eTable 3. Content of Primary Care Office-based and Telemedicine Visits, 2018-2020 (in thousands)
eFigure. Trends in Primary Care Stratified by Visit Type, 2018-2020

This supplementary material has been provided by the authors to give readers additional information about their work.

© 2020 Alexander GC et al. *JAMA Network Open.*

eTable 1. States Comprising Subnational Regions in the IQVIA National Disease and Therapeutic Index.

East South Central	Kentucky, Tennessee, Mississippi, Alabama
West South Central	Oklahoma, Arkansas, Texas, Louisiana
Mountain	Montana, Idaho, Wyoming, Nevada, Utah, Colorado, Arizona, New Mexico
South Atlantic	West Virginia, Delaware, Maryland, Washington DC, Virginia, North Carolina, South Carolina, Georgia, Florida
East North Central	Wisconsin, Michigan, Illinois, Indiana, Ohio
West North Central	North Dakota, Minnesota, South Dakota, Nebraska, Iowa, Kansas, Missouri
Pacific	Washington, Oregon, California
New England and Mid Atlantic	Maine, Vermont, New Hampshire, Massachusetts, Rhode Island, Connecticut, New York, Pennsylvania, New Jersey

Source: IQVIA National Disease and Therapeutic Index, 2018-2020

© 2020 Alexander GC et al. *JAMA Network Open.*

eTable 2. Characteristics of Primary Care Office-based and Telemedicine Visits, 2018-2020 (in thousands).

	Office-based Visits			Telemedicine Visits		
	2018 (Q1/Q2)	2019 (Q1/Q2)	2020 (Q1/Q2)	2018 (Q1/Q2)	2019 (Q1/Q2)	2020 (Q1/Q2)
Total visits (95% CI)	230,864 (221,214-240,514)	239,085 (228,733-249,437)	164,579 (157,189-171,969)	2,592 (2,015-3,169)	2,966 (2,282-3,650)	39,838 (37,021-42,655)
Age, years, n (%)						
Less than 19 (0-18)	74,583 (32.3%)	73,885 (30.9%)	54,276 (33.0%)	256 (9.9%)	590 (19.9%)	6,203 (15.6%)
19-35	29,488 (12.8%)	31,669 (13.2%)	20,328 (12.4%)	380 (14.7%)	479 (16.1%)	7,089 (17.8%)
36-55	45,580 (19.7%)	50,784 (21.2%)	32,598 (19.8%)	826 (31.9%)	538 (18.1%)	10,390 (26.1%)
56-65	31,565 (13.7%)	31,376 (13.1%)	21,350 (13.0%)	479 (18.5%)	415 (14.0%)	6,066 (15.2%)
66 or older	49,640 (21.5%)	51,361 (21.5%)	36,001 (21.9%)	651 (25.1%)	944 (31.8%)	10,090 (25.3%)
Not reported	7 (0.003%)	9 (0.004%)	27 (0.016%)	N/A	N/A	N/A
Sex, n (%)						
Male	116,073 (50.3%)	121,953 (51.0%)	83,920 (51.0%)	1,332 (51.4%)	1,494 (50.4%)	19,057 (47.8%)
Female	114,791 (49.7%)	117,132 (49.0%)	80,659 (49.0%)	1,260 (48.6%)	1,472 (49.6%)	20,781 (52.2%)
Payor, n (%)						
Commercial	138,881 (60.2%)	144,711 (60.5%)	99,249 (60.3%)	1,534 (59.2%)	1,620 (54.6%)	22,828 (57.3%)
Medicaid	37,073 (16.1%)	38,372 (16.0%)	27,225 (16.5%)	262 (10.1%)	191 (6.4%)	5,214 (13.1%)
Medicare	44,107 (19.1%)	45,465 (19.0%)	31,442 (19.1%)	533 (20.6%)	862 (29.1%)	9,080 (22.8%)
Other	10,803 (4.7%)	10,538 (4.4%)	6,663 (4.0%)	262 (10.1%)	294 (9.9%)	2,716 (6.8%)

CI confidence intervals; N/A not applicable

Source: IQVIA National Disease and Therapeutic Index, 2018-2020

© 2020 Alexander GC et al. *JAMA Network Open.*

eTable 3. Content of Primary Care Office-based and Telemedicine Visits, 2018-2020 (in thousands).

	Quarterly Office-Based Visits			Quarterly Telemedicine Visits		
	2018Q2/2019Q2	2020 (Q1)	2020 (Q2)	2018Q2/2019Q2	2020 (Q1)	2020 (Q2)
Total visits (95% confidence intervals)	117,797 (112,696-122,898)	105,911 (101,156-110,666)	58,668 (55,271-62,065)	1,403 (913-1,893)	4,794 (3,879-5,709)	35,044 (32,566-37,522)
Blood pressure recorded	88,578 (75.2%)	75,458 (71.2%)	40,873 (69.7%)	97 (6.9%)	394 (8.2%)	3,356 (9.6%)
Cholesterol assessed	27,488 (23.3%)	22,486 (21.2%)	12,681 (21.6%)	129 (9.2%)	317 (6.6%)	4,732 (13.5%)
New medicines initiated	53,664 (45.6%)	49,915 (47.1%)	26,319 (44.9%)	478 (34.1%)	1,858 (38.8%)	13,760 (39.3%)
Medicines continued	37,434 (31.8%)	33,325 (31.5%)	19,182 (32.7%)	590 (42.1%)	2,216 (46.2%)	15,439 (44.1%)
New treatment visits...						
Hypertension	3,360 (2.9%)	2,697 (2.5%)	1,615 (2.8%)	54 (3.8%)	17 (1.3%)	463 (1.3%)
Diabetes	1,404 (1.2%)	1,215 (1.1%)	705 (1.2%)	4 (0.3%)	11 (0.2%)	472 (1.3%)
High cholesterol	1,268 (1.1%)	1,326 (1.3%)	654 (1.1%)	6 (0.4%)	0 (0%)	272 (0.8%)
Asthma	1,262 (1.1%)	1,124 (1.1%)	342 (0.6%)	4 (0.3%)	22 (0.5%)	293 (0.8%)
Depression	183 (0.2%)	145 (0.1%)	101 (0.2%)	10 (0.7%)	12 (0.3%)	48 (0.1%)
Insomnia	396 (0.3%)	437 (0.4%)	95 (0.2%)	0 (0%)	0 (0%)	204 (0.6%)

Values represent average second quarter visit volume (2018-2019) and quarterly visit volume (2020Q1 and 2020Q2)

Source: IQVIA National Disease and Therapeutic Index, 2018-2020

© 2020 Alexander GC et al. *JAMA Network Open.*

eFigure. Trends in Primary Care Stratified by Visit Type, 2018-2020.

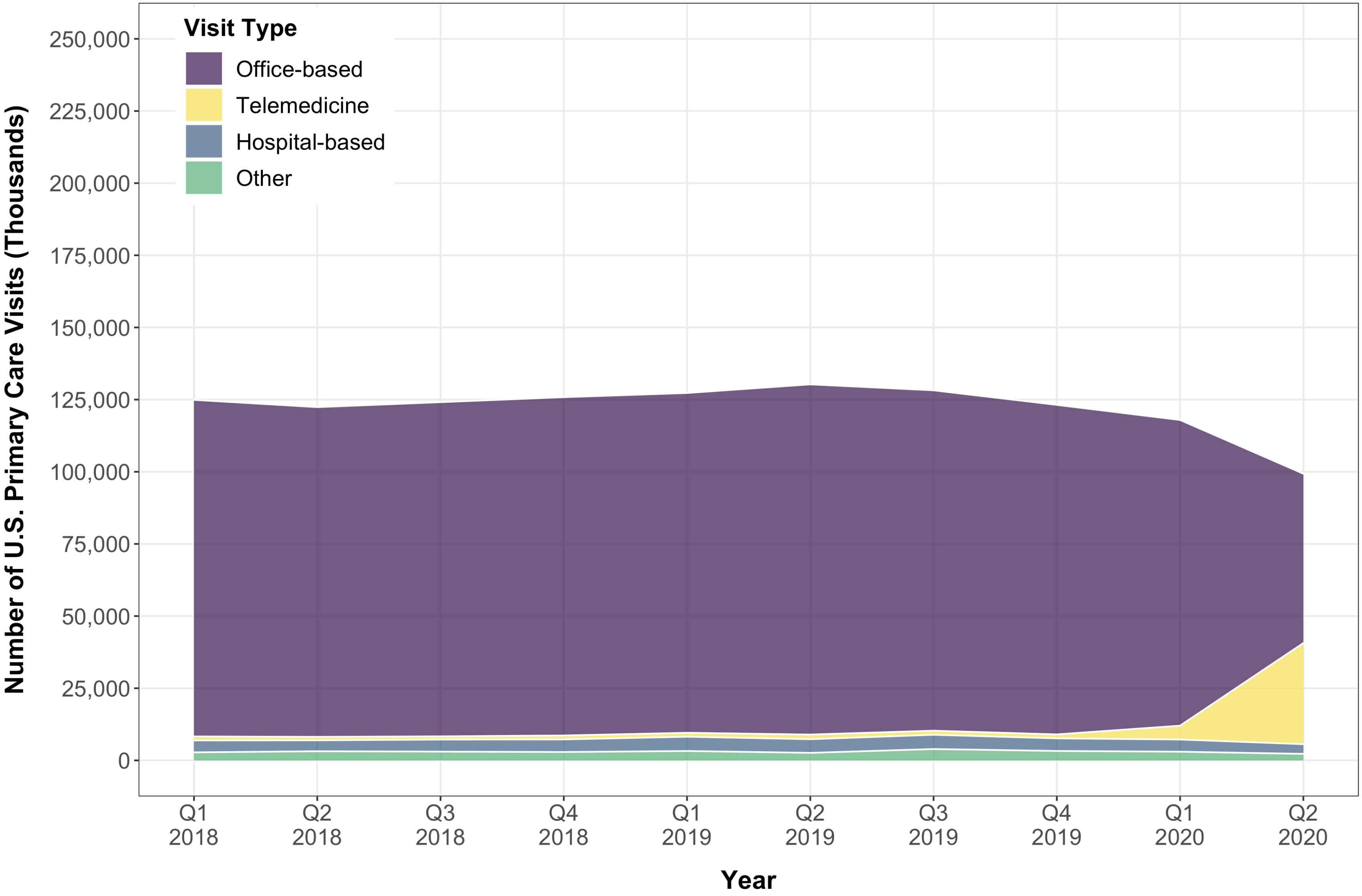

© 2020 Alexander GC et al. *JAMA Network Open.*
